# Diversifying the Interventional Cardiology Workforce: Why It Matters and How to Achieve It

**DOI:** 10.1016/j.jscai.2025.104011

**Published:** 2025-10-30

**Authors:** Anezi Uzendu, Triston B.B.J. Smith, Jason Galo, Deepali Tukaye, Yolanda Hendley, Zarina Sharalaya, Shrilla Banerjee, Jason F. Deen, Ki Park, Melvin Echols, Quinn Capers

**Affiliations:** a Division of Cardiology, Department of Internal Medicine, UT Southwestern Medical Center, Dallas, Texas; b Division of Cardiology, Department of Medicine, Veterans Affairs North Texas Health Care System, Dallas, Texas; c Cardiovascular Service Line, Trinity Medical Center West, CommonSpirit Health, St. Clairsville, Ohio; d Section of Interventional Cardiology, Medstar Washington Hospital Center, Washington, DC; e Cardiology and Vascular Medicine, Phoenix Hearts, Houston, Texas; f Cardiology, Trinity Health Mid-Atlantic Medical Group, Wilmington, Delaware; g Division of Cardiovascular Medicine, North Texas Heart Center, Medical City Heart Hospital, Dallas, Texas; h Department of Cardiology, Surrey and Sussex Healthcare NHS Trust, Redhill, Surrey, United Kingdom; i Division of Cardiology, Department of Pediatrics, University of Washington, Seattle, Washington; j Division of Cardiology, Department of Medicine, University of Washington, Seattle, Washington; k Divison of Cardiology, Department of Medicine, Morehouse School of Medicine, Atlanta, Georgia; l Department of Medicine, Howard University College of Medicine, Washington, DC

**Keywords:** bias, disparities, diversity, health equity, mentoring

## Abstract

Health care professionals continue to observe disparities in interventional cardiology associated with patient race, sex, socioeconomic status, and rurality, and the lack of diversity in the specialty is likely a major contributor. A diverse workforce will improve access to care, patient satisfaction, treatment plan adherence, clinical outcomes, and the education of a culturally sensitive workforce. However, the current sociopolitical climate is challenging the rationale supporting a diverse workforce and the methods employed to achieve it. We maintain that enhancing diversity is a rate-limiting step to reducing disparities in care. In this review, we discuss current health care inequities, the evidence that diversity will improve equitable care, barriers to enhancing diversity, and strategies to promote a diverse interventional cardiology workforce.

## Introduction

Interventional cardiology (IC) remains one of the least diverse specialties in the US. As of 2016, African Americans, Hispanic Americans, and Native Americans—groups traditionally considered “underrepresented in medicine” (URM), who together comprise 32% of the population—account for only 7.8% of board-certified interventional cardiologists and 9% of IC trainees.^1^ In contrast, URM make up 15.7% of internal medicine trainees.^2^ Women are also underrepresented within IC. Only 4.9% of board-certified interventional cardiologists and 14.5% of fellows are women, despite women constituting half the US population. For structural interventions, an analysis of the 2018 Medicare provider database, demonstrated that of the 1806 physicians who performed more than 10 transcatheter aortic valve replacement (TAVR) procedures, only 3.9% were women, and women made up just 2.7% of all interventional cardiologists performing TAVR.^3^ For comparison, in surgery, 29.1% of the active physicians are women, and 47% of the surgical residents are women.^4^

This lack of representation is not just a workforce issue; it affects access to care for underserved communities, limits culturally competent care in communities that already face a disproportionate burden of cardiovascular diseases, and perpetuates disparities in cardiovascular care.^5^ The current state and trajectory of workforce diversity and health care disparities are inconsistent with the stated missions and core values of leading cardiovascular societies. The Association of Black Cardiologists (ABC) has a mission to “… achieve health equity for all through the elimination of disparities,” The core values of the American College of Cardiology (ACC) include “patient centeredness, excellence and equity,” and the core values of the Society for Cardiovascular Angiography & Interventions (SCAI) include “inclusiveness; integrity; and leadership.” Despite the consensus that equitable care for all is a fundamental tenet of cardiology, health care disparities persist. This discussion will briefly review the current state of health care disparities in IC, describe the association between disparities and the lack of diversity in the workforce, and argue that diversifying the workforce is one strategy to eliminate disparities. We will conclude by discussing strategies to enhance diversity in IC.

## Current racial, sex, and socioeconomic disparities in coronary interventions

There are notable gaps in high-quality IC care for Black patients, including delays in door-to-balloon and door-to-drug times during ST-elevation myocardial infarctions^6^ and a reduced likelihood of being treated with drug-eluting stents when indicated.^7^ Black patients with mild to moderate chronic kidney disease are less likely to undergo invasive management for acute myocardial infarction compared to White patients with similar renal function.^8^ A contemporary review of Medicare data showed that Black race was associated with a lower likelihood of receiving extracorporeal membrane oxygenation support in the setting of cardiogenic shock, despite being treated at a large hospital with advanced mechanical support programs.^9^ The potential drivers of these differences are numerous, but they are likely influenced by a higher burden of adverse social determinants of health in Black communities, structural biases of institutions, racial differences in health insurance, explicit and implicit biases of health care providers, and decreased health literacy of individuals from underserved communities.^10^, ^11^, ^12^ There is also well-founded mistrust of medical institutions in some Black communities due to historic and current mistreatment, which leads individuals to delay seeking care and can result in poor adherence to treatment plans.

Heart disease accounts for more deaths in women than any other disease, with a significant percentage of those deaths due to acute coronary syndromes (ACS).^13^ Women with ACS are less likely than men to be treated with percutaneous coronary intervention or even angiography when they present for evaluation.^14^, ^15^, ^16^ This may partially explain why women have higher in-hospital mortality and complications when presenting with ST-elevation myocardial infarction compared to men.^17^ Although women may have atypical symptoms more often than men and present later to the hospital,^14^ when they do present, they are more likely to be misdiagnosed or receive a less thorough evaluation.^18^ Women with ACS are also older and have a higher burden of comorbidities when evaluated,^19^ which may influence cardiologists to select femoral vs radial artery access approaches or avoid invasive therapies altogether due to the perception that women are frail, have smaller radial arteries, and are more likely to have a complicated post–percutaneous coronary intervention course. Additionally, the fact that women are underrepresented in clinical trials results in a lack of robust data on the best interventional therapies for women. The combination of these factors explains some of the disparities in diagnosis, management, and outcomes seen by sex.

Rural counties in the US are nearly 5 times less likely to have available diagnostic and interventional cardiac catheterization services.^20^ A separate analysis showed that patients hospitalized for ACS who live in an area with a low density of cardiologists have a higher 1-year mortality.^21^ Although 1 in 5 Americans live in rural areas, around 1 in 10 physicians practice there, largely due to challenges in recruiting and retaining specialists like cardiologists. The shortage of interventional cardiologists in rural and underserved areas is likely multifactorial^22^ and addressing it should be an area of focus for training hospitals and health systems. Improving access by developing telehealth infrastructures, increasing the pipeline for trainees from rural communities, offering incentives for physicians who decide to serve rural communities, and hospital sponsorships of visa waiver programs for international medical graduates are a few strategies that are being proposed to increase the number of interventional cardiologists in rural counties.^23^^,^^24^ Another challenge for IC care in small rural counties is the need to maintain procedural volume to optimize outcomes. Regionalized systems of care, such as “hub and spoke” models with outreach clinics/hospitals, may allow access to life-saving therapies while maintaining quality.

## Current racial, sex, and socioeconomic disparities in structural heart interventions

Black, Hispanic, American Indian, and Alaska Native patients and women are less likely to receive structural heart disease therapies despite advances in these life-saving procedures. Black patients account for only 4% of TAVR recipients in the US.^25^ Hispanic patients are similarly underserved, and American Indian and Alaska Native populations face even greater barriers, with some data suggesting near-zero access to TAVR in tribal areas.^26^ Several factors are likely to drive these disparities, such as referral biases, higher rates of patients declining the procedures, and socioeconomic and language barriers.^27^

Women treated with structural heart interventions have lower postoperative mortality at 1 year compared to male counterparts, yet they are often undertreated and experience significant delays prior to intervention compared to men.^28^^,^^29^

Patients with low income are significantly less likely than medium- and high-income patients to have a variety of structural heart disease procedures, including TAVR, implantation of left atrial appendage occlusion devices, and transcatheter mitral valve repair procedures.^25^^,^^30^^,^^31^ Adverse social determinants of health and biases in initial diagnosis, care delivery, and referral patterns are among the drivers of these disparities.^32^

## Diverse workforce as one strategy to reduce disparities in IC care

Solutions to address health care disparities in IC include but are not limited to health care payment reform, recognition and mitigation of individual and structural biases, shared decision-making with patients, investment in community engagement efforts, oversight by regulatory agencies for equitable uptake of novel therapies, and partnerships between tribal leadership and community committees. There are multiple comprehensive reviews on solutions for cardiovascular disparities.^12^^,^^33^ Our focus is to review the evidence that a diverse IC workforce is key to advancing health care equity in the specialty.

The contention that a diverse workforce will reduce health care disparities is supported by several lines of evidence. Despite being a small proportion of the workforce, Black, Hispanic, and Asian physicians are “the usual care source” for 53% of minority and 70% of non-English speaking patients, based on an analysis of the nationally representative Medical Expenditure Panel Survey. The same analysis found that patients defined as earning low incomes or who have Medicaid insurance are more likely to have a minority rather than a White physician.^34^ Race concordance between physicians and patients has been associated with enhanced doctor-patient communication,^35^ improved patient ratings of their experiences,^36^ and increased adherence to physicians’ recommendations.^37^ Recent studies extend the positive implications of a diverse physician workforce to improved survival. Medical^38^ and surgical^39^ patients may have improved survival when treated by women vs men, and Black individuals residing in counties with higher concentrations of Black primary care physicians experience a longer lifespan^40^ and a lower Black-White mortality gap. Considering medical education and training, White students in racially diverse classrooms rate themselves as better prepared to care for diverse patients than their counterparts at less diverse schools.^41^ Finally, with regard to research, interventional cardiologists from underrepresented backgrounds are more likely to study and call attention to health inequities that might otherwise be overlooked. For instance, research on disparities in structural heart interventions has been driven primarily by investigators from diverse backgrounds.^25^ These insights can shape guidelines and expand the reach of interventional therapies. Thus, a diverse IC workforce can be expected to improve patient outcomes by each of the following mechanisms: increasing outreach and service to medically underserved and disadvantaged communities, improving communication and trust with groups traditionally wary of the health care system, improved cultural competency and sensitivity of all physicians, and by producing research that includes diverse populations and illuminates solutions for health care disparities.

## Current barriers to increasing diversity in the workforce

### Adverse social determinants of success (poverty, racial bias, and discrimination in early grades)

Obstacles to diversity in the IC workforce can exist in the early experiences of children from underrepresented groups. Social determinants of success, such as poverty, educational inequities, and racial bias during early grades, play a significant role in shaping academic trajectories. These disadvantages accumulate over the years and decrease the number of Hispanic, Black, and Native American students who emerge from high school prepared to pursue pre-med programs in college or navigate subspecialties of medicine.^42^ Also, the burden of student loan debt may deter medical students from lower-income backgrounds from considering IC due to the additional years of training required.

### Barriers specific to work-life balance and childbearing

Women internal medicine residents cite concerns about radiation during childbearing years, the physically demanding nature of the job, the lack of women role models, and gender discrimination as factors making IC less attractive.^43^ Mentorship from women with productive and healthy careers in IC will be critical to dispel such reservations. The demands of being on-call and working extended hours may deter physicians who desire a healthy work-life balance from pursuing the specialty. Education and increased awareness about the diversity of practice settings and schedules will be critical to keep interest high among young doctors. Senior interventional cardiologists should discuss these topics and speak about their experiences as successful physicians with satisfying lives outside of medicine. Organizations like ACC, SCAI, the ABC, and others can be effective conduits for communicating this information via educational programs. Unlike for women, there is a current knowledge gap in the published literature regarding how URM view IC. It is our hope that our cardiology organizations will sponsor and publish such a survey.

### The US Supreme Court decision on affirmative action and “anti-DEI” political sentiment

In 2023, the Supreme Court of the United States ruled that it is unconstitutional to consider race in higher education admissions.^44^ This will complicate efforts to actively recruit individuals from underrepresented groups into college and medical school. Though not specifically addressing recruitment into residency and fellowship training programs, the Court’s decision, combined with a recent wave of anti–diversity, equity, and inclusion (anti-DEI) state laws^45^ and the issuance of an anti-DEI Executive Order by the US President in 2025,^46^ is likely to negatively impact efforts to foster diversity in graduate medical education (GME) training programs and in the profession of medicine and its subspecialties.^47^

### Bias in the selection processes along the educational pathway

Children from URM backgrounds who aspire to become physicians must overcome nearly 2 decades of structural and individual biases that limit their upward mobility in the education pipeline. This is evidenced by articles that show a racial bias in referring Black and Hispanic children to gifted and talented programs in the early school grades,^48^ others that reveal an anti-Black bias in grading writing samples of high school students,^49^ and one study revealing that faculty serving on medical school admissions committees might have an unconscious negative bias against Black people.^50^ Finally, several studies have found evidence of racial bias in the selection process for GME training positions.^51^ The lack of diversity in IC is unlikely to improve in the coming decades if individual and structural biases in the educational pathway to medicine remain unchecked.

## Strategies to increase diversity in the IC workforce

### Increasing the supply of talented underrepresented individuals in the educational pathway

#### Elementary to premedical students

Expanding access to quality early childhood education programs and creating longitudinal mentorship programs are key strategies for addressing hurdles to advancement in the educational pathway. Cardiology organizations could lobby for improvements in early childhood education and should continue to sponsor programs that offer exposure to medical careers.

For secondary and postsecondary students who aspire to be physicians, exposure to physician mentors is critical to remain focused on the long path from elementary school to a career in medicine. Examples of community-based programs that expose children to the career of medicine include “Young Doctors DC,” “Young Physicians Initiative,” and “Made for Medicine.”^52^^,^^53^ Many academic medical centers support programs for precollege students, like The Ohio State University College of Medicine’s “MD Camp” that exposes high school students to clinical shadowing and medical student mentors.^54^ Professional societies like the ABC, the American Heart Association,^55^ and the ACC^56^ sponsor a variety of programs that encourage high school and college students to pursue careers in cardiology. Programs that expose children and young adults to medical careers and, importantly, to minority and women physicians, need to be supported and replicated many times over.

Another potential positive is the recent proliferation of tuition-free education for some undergraduate and medical schools. This could relieve significant financial barriers to pursuing careers in medicine for talented students from less privileged backgrounds. However, it remains to be seen whether these programs will result in a more diverse physician workforce.

#### Medical students

For minority and women medical students who may perceive IC as inaccessible, practical experiences can demystify the field. The ACC Medical Students Member Community exposes medical students to the clinical and procedural aspects of cardiology, and SCAI’s “Ready to Launch”^57^ program provides an in-depth exposure to the subspecialty of IC. Held in conjunction with the SCAI scientific conference, it provides hands-on experiences with interventional equipment and networking opportunities with a racially diverse group of interventional cardiologists. These initiatives do not exclude participants from any demographic group and have the potential to build an IC workforce poised to deliver culturally responsive care and reduce cardiovascular disparities.

#### GME trainees

For GME trainees, several issues require attention. For women, engaging with female IC specialists is critical, but because of the demographic characteristics of the specialty, it is unlikely at many centers. Also, due to program constraints, many internal medicine residents may not have access to an interventional cardiac catheterization laboratory where they can shadow or observe. The ACC Internal Medicine Cardiology Program, initiated in 2020, connects residents with cardiologist mentors and provides a curriculum that educates them about cardiology and its subspecialties via webinars and panel discussions with cardiology program directors.^58^ Through this and similar programs, women training in internal medicine interact with women interventional cardiologists who can serve as mentors, and all participating residents engage with practicing interventional cardiologists virtually and in-person at the annual ACC Scientific Sessions meeting.

For years, selecting IC fellows exposed applicants to a system in which each program utilized unique applications and deadlines and required applicants to respond to offers immediately. This seemed to put international medical graduates or cardiology fellows who identify as Hispanic, Black, or Native American at a disadvantage, and the same is true for applicants who train in community hospitals or university medical centers that are perceived to be less prestigious. The initiation of an IC Match through the National Resident Matching Program in the 2024-2025 cycle has been a welcome advance and is expected to standardize the process and level the playing field for candidates who have traditionally faced challenges securing IC training positions.

### Counteracting bias in the selection processes

Becoming an IC requires the completion of years of higher education, a minimum of 6 years of postgraduate clinical training, and the mastery of advanced invasive cardiovascular techniques in an IC fellowship. At several steps in this process, the trainee is evaluated by a group of individuals who decide whether they will advance to the next stage. Biases can influence the process as well as the selection committee members.

An example of the potential for bias in the process is the use of unstructured interviews, in which candidates are asked nonstandardized questions by interviewers with subsequent questions depending on the candidate’s responses. Unstructured interviews are more prone to being impacted by biases of the interviewers, and questions may not accurately assess candidate qualifications. To mitigate the impact of biases in the selection of medical students, residents, and fellows, we advocate for structured interviews^59^^,^^60^ in which all interviewees respond to a standard set of questions that are evaluated according to a prespecified rubric and implicit bias mitigation training for selection committees.^61^^,^^62^

Other possible bias mitigation strategies include recruiting a diverse faculty/selection committees and redacted/blinded application screening and scoring. A recent publication found that GME programs with more women tended to attract and match more women applicants and programs with more minority faculty tended to match more minority applicants.^63^ The merits of redacting applicants’ race, ethnicity, and gender from the application as a strategy to mitigate the impact of potential racial or gender biases are less certain. A few small studies assess this strategy: one found no difference in applicant scores when identifiers were redacted,^64^ whereas another found that blinded interviewers ranked URM applicants higher than nonblinded interviewers.^65^ We await further studies to better inform this practice.

### Selecting for potential to be an advocate of health equity in IC

Interventional cardiology needs clinicians and academicians who will lead efforts to ensure that current and future technologies are deployed in an equitable manner. Just as training programs with a mission to graduate physician-scientists use achievements in research as criteria for selection, programs that aim to produce interventional cardiologists committed to reducing health care disparities should recruit physicians with a strong record of prioritizing health equity and service to the underserved. Applicants who have provided health care to disadvantaged communities, completed scholarly projects on the topic of health care disparities, or led community service and empowerment efforts are more likely to champion health equity than those who have not. We previously published a checklist that considers the applicant’s “health equity competency” along with traditional characteristics, such as clinical and technical skill, collegiality, leadership, and academic curiosity, and recommend it as a tool to help recruit fellows from all backgrounds who have the potential to promote health equity in our field.^66^

### Fostering impactful mentorship

Mentorship is particularly crucial for URM and female trainees. Race and/or gender concordance between mentors and mentees has been associated with improved outcomes,^67^ but it is not practical to expect that all mentorship pairs will have this concordance. We propose that all early career interventional cardiologists (trainees and attendings alike) be assigned a mentor by their employer, and that all mentors receive formal training in the best practices to support and advise mentees, which has been shown to improve outcomes.^68^

There are mentorship programs available through the ACC/American Heart Association, SCAI, British Cardiovascular Society, and WomenAsOne with opportunities for trainees at different stages of an academic career. With the rise of social media and virtual platforms, mentorship pairings can extend beyond geographic barriers and expose trainees or junior faculty to role models in distant locations.

## Summary

Racial and gender disparities in interventional cardiovascular care have been documented for at least 30 years, and the field has lacked racial and gender diversity since its founding. Years of describing both problems have not led to significant improvements. Focusing on the following evidence-based strategies will increase diversity in our field: (1) enriching the academic pathway with people from groups underrepresented in the specialty; (2) mitigating bias in the selection processes at each step in the pathway; (3) prioritizing health equity competency as a selection metric; and (4) mentoring aspiring interventional cardiologists, trainees, and young faculty. Table 1^43^, ^44^, ^45^, ^46^^,^^48^^,^^50^^,^^51^^,^^54^^,^^55^^,^^61^^,^^66^^,^^68^ lists barriers and strategies to enhancing diversity in IC with appropriate citations.

**Table 1:** Barriers to enhancing diversity and strategies to enhance diversity in IC (with references from the article)

Barriers to increasing diversity in IC	Illustrative examples	Strategies to increase diversity in IC	Illustrative examples
Adverse social determinants of success	• Racial discrimination in early grades^48^ • Student loan debt in low income families	Increasing the supply of talented underrepresented individuals early in the pipeline	• The Ohio State University “MD Camp”^54^ • AHA HBCU Scholars program^55^
Negative perceptions about radiation during childbearing years and a difficult work-life balance	• Survey of women residents^43^	Counteract bias in selection processes	• Bias mitigation training for selection committees^61^
Anti-DEI, antiaffirmative action political and judicial climate	• SCOTUS ruling against affirmative action in education admissions^44^ • Proliferation of anti-DEI state laws^45^ • Presidential anti-DEI order^46^	Prioritize health equity competency when recruiting/ranking candidates	• Recruit physicians with a track record of serving underserved populations and advocating for health equity in IC^66^
Bias in selection processes	• Implicit racial bias in medical school admissions^50^ • Racial bias in GME selections^51^	Promote impactful mentorship of trainees and junior faculty	• Employers assign IC mentors to all IC trainees and early career physicians • Mentors train in best practices^68^

AHA, American Heart Association; DEI, diversity, equity, and inclusion; GME, graduate medical education; HBCU, Historically Black Colleges and Universities; IC, interventional cardiology; SCOTUS, Supreme Court of the United States.

As the field of IC approaches its 50th birthday, we will soon celebrate its extraordinary evolution from treating single-vessel coronary artery disease with balloon angioplasty to life-saving procedures that treat complex coronary, vascular, and structural heart disorders. We must ensure that all populations and demographic groups benefit from the innovative therapies in our field. One important strategy to achieve that goal is to significantly increase the diversity in the specialty.
